# An Evaluation of the Effectiveness of Sorbents in the Remediation of Soil Contaminated with Zinc

**DOI:** 10.1007/s11270-018-3882-2

**Published:** 2018-07-01

**Authors:** Rafał Strachel, Jadwiga Wyszkowska, Małgorzata Baćmaga

**Affiliations:** Department of Microbiology, University of Warmia and Mazury in Olsztyn, Plac Łódzki 3, 10-727 Olsztyn, Poland

**Keywords:** Pollution, Sorbents, Remediation of soil, Microorganisms, Enzymes, Biodiversity

## Abstract

Zinc exerts negative effects on soil and contributing to the degradation of soil ecosystems. New solutions for restoring healthy soil activity are therefore needed. The aim of this study was to evaluate the effectiveness of sorbents in the biological remediation of soil contaminated with zinc. A pot experiment was conducted on loamy sand. The tested plant was maize (*Zea mays*). Soil was contaminated with zinc chloride doses of 0, 100, 300, and 900 mg Zn^2+^ kg^−1^ DM soil (dry matter of soil). Alginate, biochar, sepiolite, calcined halloysite, and a molecular sieve were added to soil in amounts corresponding to 2.5% of soil weight to minimize zinc’s potentially toxic effects on the biological properties of soil. The application of zinc stimulated the proliferation of all analyzed microbial groups. Zinc exerted negative effects on the ecophysiological diversity (EP) of fungi and the activity of dehydrogenases, catalase, and acid phosphatase. The applied sorbents modified the microbiological and biochemical properties of soil. In zinc-contaminated soil, alginate, biochar, and molecular sieve increased the counts of organotrophic, oligotrophic, and actinobacteria. Sorbents were not highly effective in promoting fungal growth and exerted varied effects on the activity of soil enzymes. The molecular sieve stimulated the activity of all soil enzymes, excluding β-glucosidase. Alginate minimized the negative influence of zinc on dehydrogenases and acid phosphatase, and biochar—on catalase, sepiolite, and calcined halloysite —on acid phosphatase. By modifying the biological properties of soil, the tested sorbents contributed to an increase in maize yields and a decrease in zinc uptake by maize plants.

## Introduction

Over the decades, human activities have contributed to a disruption in soil biological activity. Environmental pollution caused by chemical compounds, including heavy metals, exerts negative effects on ecosystems and crop production (Mahar et al. 2016). Heavy metals are persistent pollutants, and they pose a problem in both urban and rural areas when their accumulation in soil exceeds safe levels. The quantity of trace elements in the environment does not change over time, and these elements can only be transformed into biologically available or unavailable forms (Khalid et al. 2017). The availability of heavy metals in soil is closely associated with the physicochemical properties of soil. Biologically available heavy metals are present in the soil solution or they occur as exchangeable fractions bound to organic or inorganic soil components. Metals that are not available to plants are strongly bound to soil particles; they are present in the crystal lattice of soil minerals or form insoluble deposits. Changes in the physicochemical properties of soil, i.e., pH, cation-exchange capacity, content of organic matter, clay minerals, Fe, Al, and Mn oxides, and redox potential, can lead to the transformation of biologically unavailable compounds into plant-available forms (Aydinalp and Marinova 2003).

Zinc is an essential element for living organisms (Krężel and Maret 2016). However, elevated zinc concentrations can disrupt the biological balance (Strachel et al. 2017). Excessive accumulation of zinc in soil can deteriorate the physicochemical and biological properties of soil, which leads to soil degradation and renders soil unfit for agricultural production (Wyszkowska et al. 2016). In zinc-contaminated habitats, effective measures are required to restore the biological functions of soil. Soil remediation is one of the most popular solutions that address this problem. Pollutants can be extracted from soil, but the relevant measures are costly and time-consuming. Soil pollutants can also be stabilized with the use of various substances that decrease the mobility of heavy metals. Solutions applied in situ are less expensive and can be applied over larger areas of contaminated land (Pérez de Mora et al. 2005).

The use of organic and inorganic adsorbents to stabilize the chemical composition of soil contaminated with heavy metals is an economical and environmentally friendly remediation method. Adsorbents immobilize heavy metals and minimize their adverse effects on living organisms, in particular soil-dwelling microorganisms and plants (Malandrino et al. 2011).

Various organic substances are increasingly often used to reduce the availability of metals in soil and minimize the adverse environmental impact of zinc. These substances reduce the mobility of contaminants by binding chemical elements or modifying local environmental conditions to decrease their solubility (Bradl 2004). The influence of sorbents with remediation potential on zinc-contaminated soil was investigated in this study. The effectiveness of alginate, biochar, sepiolite, calcined halloysite, and a molecular sieve was evaluated in soil cotaminated with zinc. Due to the complexity of the soil environment, the remediation potential of the tested sorbents was analyzed based on changes in the counts and diversity of soil-dwelling microorganisms, changes in the biochemical activity and physicochemical properties of soil, and the development of maize plants.

## Materials and Methods

### Soil

The experiment was performed on brown calcic soil developed from loamy sand. Soil was sampled at the area north-eastern Poland (53.7161° N, 20.4167° E). The properties of the experimental soil have been described in detail in a previous study Strachel et al. (2017).

### Sorbents

Five sorbents were evaluated in a pot experiment: alginate, biochar, sepiolite, calcined halloysite, and a molecular sieve.

Alginate was manufactured by Vázsony-Szövetkezeti Kft. (Hungary). This mineral compound is a rich source of nutrients. According to label data, the product contains (g kg^−1^): humic substances—200–300, Ca—100–300, N—3–5, P—5–6, K—6–9, and Mg—8–10. Alginate also contains trace amounts of Mn, Cu, Zn, Fe, B, and Mo. The pH values of alginate are pH_KCl_—6.7 and pH_H2O_—7.2.

Biochar was manufactured by Fluid company (Poland). The product is made from organic material via pyrolysis. Biochar has the following chemical composition: stable C—more than 77%, volatile matter —17%, ash—less than 6%, chlorides, sulfur, and mercury—less than 0.01%. The pH values of biochar are pH_KCl_—8.0 and pH_H2O_—9.0.

Sepiolite 60/100 contains sepiolite (Mg_4_[Si_6_O_15_(OH)_2_]6H_2_O), and it was manufactured by the Sepiolsa Minersa Group (Spain). Sepiolite is hydrated magnesium silicate with adsorptive properties. According to label data, the product contains 70% sepiolite, including 85% of particles with the size of 75 to 710 μm. The pH values of sepiolite are pH_KCl_—7.1 and pH_H2O_—7.1.

Halloysite (Al_2_Si_2_O_5_(OH)_4_) is a clay mineral which is used in the production of Halosorb mineral sorbents (Intermark, Poland). The mineral used in this experiment was mined in Dunino (Poland), and it has the following chemical composition: SiO_2_—40 ± 1%, Fe_2_O_3_/FeO—8 ± 1%, TiO_2_—2 ± 1%, Al_2_O_3_—33 ± 1%, MgO—0.5 ± 0.1%, CaO—1.3 ± 0.2%, Na_2_O—0.1%, and K_2_O—0.1%. Halloysite is characterized by a large specific surface area, high porosity, and high cation-exchange capacity. Halloysite was calcined before the experiment. The pH values of halloysite are pH_KCl_—5.9 and pH_H2O_—6.5.

The Sylosiv A3 molecular sieve was manufactured by Grace Davison Company (USA). This synthetic nanoporous material consists of crystalline aluminosilicate with 0.3-nm micropores. Molecular sieve powder is a zeolite with a three-dimensional pore system and extensive adsorption capacity. It is available in the form of white odorless powder. The pH values of molecular sieve are pH_KCl_—8.5 and pH_H2O_—10.2.

### Experimental Design

A pot experiment was conducted in the greenhouse in five replications. Soil material in the amount of 3 kg of air-dried soil was placed in 3.5-dm^3^ polyethylene pots with various doses of zinc, remediation substances, and mineral fertilizers. Zinc was applied as ZnCl_2_ in doses of 0, 100, 300, and 900 mg Zn^2+^ kg^−1^ DM soil. The tested sorbents—alginate, biochar, sepiolite, calcined halloysite, and a molecular sieve—were applied to soil in amounts corresponding to 2.5% of soil weight. Four zinc doses and six types of sorbents were analyzed in five replications—a total of 120 pots. The tested plant was maize which was grown for 60 days. Water content at the level of 50% of the maximum capacity of the soil was maintained throughout the entire experimental period. The following mineral fertilization was applied (pure element per mg kg^−1^ soil): N—100, P—50, K—100, Mg—25, Cu—5, Mn—5, Mo—2.5, and В—0.33. Mineral fertilizers were applied in the following forms: nitrogen—as CO(NH_2_)_2_, phosphorus—as KH_2_PO_4_, potassium—as KH_2_PO_4_ + KCl, magnesium—as MgSO_4_·7H_2_O, copper—as CuSO_4_·5H_2_O, manganese—as MnCl_2_·4H_2_O, molybdenum–as Na_2_MoO_4_·2H_2_O, and boron—as Н_3_ВO_3_. Maize was sown at 12 seeds per pot. Seedlings were thinned to leave nine plants per pot. Maize plants were harvested in stage BBCH 53 (inflorescence emergence—tip of inflorescence visible) and dried at a temperature of 60 °C until the achievement of constant weight. Microbiological and biochemical analyses of soil were performed in triplicate for each soil sample on days 30 and 60 of the experiment. The physicochemical properties of soil were determined on day 60, in triplicate for each sample.

### Analyses

Microbial counts and enzyme activity in soil samples were determined in three replicates on experimental days 30 and 60. The physicochemical properties of soil and zinc concentrations in soil and plant material were determined on day 60.

Microbiological analyses were carried out by the plating method to determine the counts of microorganisms. The counts of organotrophic bacteria, actinobacteria, and fungi were estimated over a period of 10 days in daily intervals to determine the ecophysiological diversity (EP) index of microorganisms according to Eq. (1) (De Leij et al. 1993). The activity of dehydrogenases, catalase, urease, acid phosphatase, alkaline phosphatase, and β-glucosidase was determined in soil. Microbiological and enzymatic analyses have been described in detail in a study Borowik et al. (2017).

The activity of soil enzymes (dehydrogenases, catalase, acid phosphatase, alkaline phosphatase, urease, and β-glucosidase) was used to calculate the soil biochemical quality index (BA) based on Eq. (2) (Wyszkowska et al. 2013) and to determine soil resistance (RS) to zinc pollution according to Eq. (3) (Orwin and Wardle 2004). Microbial counts and enzyme activity were also used to calculate the zinc impact factor IF_Zn_ (Eq. 4) and the sorbent impact factor IF_s_ (Eq. 5) (Kaczyńska et al. 2015).

1 $$ \mathrm{EP}=-\sum \left(\mathrm{pi}\cdotp \log\ \mathrm{pi}\right) $$

where

EP: ecophysiological diversity index of microorganisms,
pi: share of individuals of the i^th^ species in the community relative to the total number of individuals in the community

2 $$ \mathrm{BA}=\mathrm{Deh}+\mathrm{Cat}+\mathrm{Pac}+\mathrm{Pal}+\mathrm{Ure}+\mathrm{Glu} $$

where

BA: biochemical quality index,
Deh: activity of dehydrogenases (μmol TFF kg^−1^ DM of soil h^−1^),
Cat: activity of catalase (mol O_2_ kg^−1^ DM of soil h^−1^),
Pac: activity of acid phosphatase (mmol PNP kg^−1^ DM of soil h^−1^),
Pal: activity of alkaline phosphatase (mmol PNP kg^−1^ DM of soil h^−1^),
Ure: activity of urease (mmol N-NH4 kg^−1^ DM of soil h^−1^),
Glu: activity β-glucosidase (mmol PNP kg^−1^ DM of soil h^−1^).

3 $$ \mathrm{RS}\left({t}_0\right)=1-\frac{2\left|{D}_0\right|}{C_0+\left|{D}_0\right|} $$

where

RS: soil resistance index;
*C*_0_: soil resistance under natural conditions over time *t*_0_;
*P*_0_: resistance of soil subjected to pressure over time *t*_0_; *D*_0_ = *C*_0_ – *P*_0_.

The soil resistance index (RS) varies from − 1 to 1. If the value of RS is − 1 and 0, zinc exerts a very strong effect on soil. If the value of RS approximates 1, soil is more resistant to zinc.

4 $$ \mathrm{IFZn}=\frac{A_{\mathrm{Zn}}}{A_0} $$

where

IF_Zn_: zinc impact factor,
A_Zn_: enzyme activity or microbial counts in soil contaminated with zinc,
A: enzyme activity or microbial counts in uncontaminated soil.

5 $$ \mathrm{IFZn}=\frac{A_{\mathrm{S}}}{A}\kern0.5em $$

where

IF_s_: sorbent impact factor,
A_S_: enzyme activity or microbial counts in soil with the addition of sorbents,
A: enzyme activity or microbial counts in soil without the addition of sorbents.

The physicochemical parameters of soil and the content of plant-available zinc and total zinc in soil (flame atomic absorption spectrometry method - FAAS) were determined according to Harris (2006). The detection limit was 96 μg dm^−3^ for total zinc and 13 μg dm^−3^ for bioavailable zinc. Uncertainty was estimated at a confidence level of *p* = 0.95 and coverage factor *k* = 2. To determine total zinc content, soil samples were digested in a mixture of concentrated HCl and HNO_3_. Bioavailable zinc content was determined after extraction with 1 M HCl. The distribution of zinc in plants was determined based on zinc concentrations in the aerial parts and roots of maize plants (Zacchini et al. 2009).

### Statistical Analysis

The results were processed statistically in Statistica 12.0 software (StatSoft Inc. 2014) at a significance level of *p* = 0.05. The percentage contribution of the experimental factors to the observed variation (*η*^2^) was determined. Microbial counts and enzyme activity were subjected to principal component analysis (PCA). Homogeneous groups were identified by Tukey’s HSD test.

## Results

### Microbial Properties of Soil

The results of the study (Table 1) indicate that the type of sorbent exerted the greatest influence on microbial counts, ranging from 37.17% for copiotrophic bacteria to 51.80% for organotrophic bacteria. Zinc dose induced somewhat smaller changes in microbial counts, and its influence ranged from 15.29% for organotrophic bacteria to 33.70% for copiotrophic bacteria. Microbial counts were least affected by the date of analysis (0.33 to 5.93%).

**Table 1:** Percentage contribution of the experimental factors to the observed variation (*η*^2^) in microbial counts in zinc-contaminated soil

Factors	Org	Cop	Olig	Act	Fun
Dose of Zn (a)	15.29	33.70	25.67	19.43	31.50
Sorbents (b)	51.80	37.17	43.70	45.55	54.38
Time (c)	0.33	1.97	0.33	5.93	4.25
a · b	10.49	14.12	7.68	7.21	4.42
a · c	0.52	2.37	1.28	1.35	1.94
b · c	6.47	1.05	11.66	12.52	0.98
a · b · c	11.63	6.75	8.38	5.23	1.50
Error	3.47	2.87	1.30	2.78	1.03

*Zn* zinc, *Org* organotrophic bacteria, *Cop* copiotrophic bacteria, *Olig* oligotrophic bacteria, *Act* actinobacteria, *Fun* fungi

**Fig. 1:**
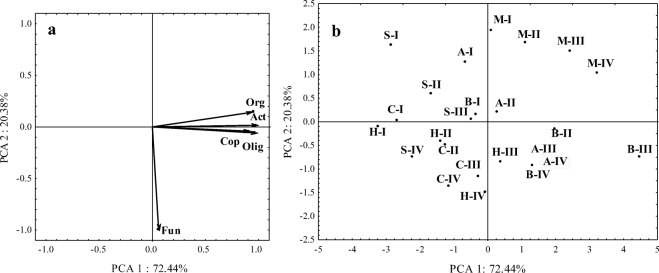
Microbial counts in zinc-contaminated soil in principal component analysis (PCA). **a** Projection of vectors onto a plane describing microbial counts. **b** Projection of cases onto the plane. Org organotrophic bacteria, Cop copiotrophic bacteria, Olig oligotrophic bacteria, Act actinobacteria, Fun fungi, dose of zinc (mg Zn^2+^ kg^−1^ DM soil): I 0, II 100, III 300, IV 900; Sorbents: C control, A alginite, B biochar, S sepiolite, H halloysite, M molecular sieve

In the principal component analysis (Fig. 1a), soil microorganisms were divided into two groups based on their responses to the experimental factors. The first group was composed of organotrophic bacteria, copiotrophic bacteria, oligotrophic bacteria, and actinobacteria. The second group consisted of fungi which responded differently to the analyzed factors. The counts of bacteria and actinobacteria were most influenced by biochar, molecular sieve, and alginate, and they were least influenced by sepiolite (Fig. 1b). The above results were inferred from the projection of cases describing treatments with the tested sorbents relative to the vectors describing microbial counts. Fungal counts were highest in treatments with zinc doses of 300 and 900 mg Zn^2+^ kg^−1^ DM soil.

**Fig. 2:**
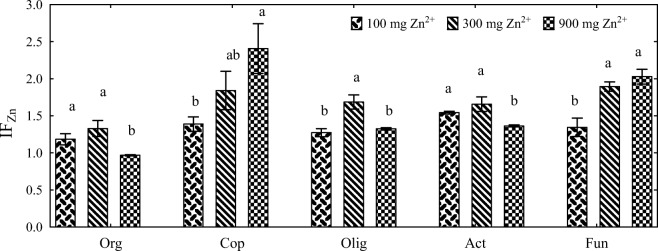
Influence of zinc (IF_Zn_) on soil microbial counts in treatments without sorbents (mean values independent of time). Org organotrophic bacteria, Cop copiotrophic bacteria, Olig oligotrophic bacteria, Act actinobacteria, Fun fungi. Homogeneous groups (a, b) were created separately for each microbial group (*n* = 9; *p* = 0.05)

The calculated values of IF_Zn_ indicate that zinc exerted varied effects on microbial counts (Fig. 2). In treatments without sorbents, increasing doses of zinc (100 to 900 mg Zn^2+^ kg^−1^ DM soil) increased the proliferation of copiotrophic bacteria, oligotrophic bacteria, actinobacteria, and fungi. The growth of organotrophic bacteria was inhibited only by the highest zinc dose (900 mg Zn^2+^ kg^−1^ DM soil). Based on the calculated values of IF_Zn_, the evaluated soil-dwelling microorganisms were arranged in the following order (from most resistant to most sensitive): copiotrophic bacteria < fungi < actinobacteria < oligotrophic bacteria < organotrophic bacteria.

**Fig. 3:**
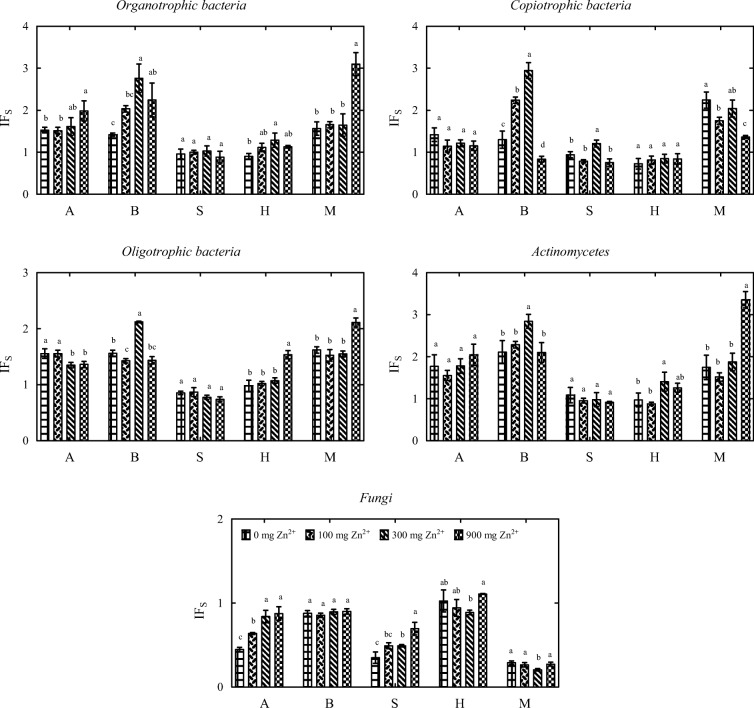
Influence of sorbents (IF_s_) on soil microbial counts (mean values independent of time). A alginite, B biochar, S sepiolite, H halloysite, M molecular sieve. Dose of zinc 0, 100, 300, and 900 mg Zn^2+^ kg^−1^ DM soil. Homogeneous groups (a–d) were created separately for each microbial group and each sorbent (*n* = 12; *p* = 0.05)

The calculated values of IF_s_ indicate that the proliferation of soil-dwelling microorganisms was also influenced by the type of sorbent (Fig. 3). In treatments not contaminated with zinc, the addition of alginate, biochar, and molecular sieve stimulated the proliferation of organotrophic bacteria, copiotrophic bacteria, oligotrophic bacteria, and actinobacteria. The tested sorbents did not stimulate the growth and development of fungi whose IF_s_ values ranged from 0.290 (molecular sieve) to 1.022 (halloysite). Alginate, biochar and molecular sieve stimulated the proliferation of organotrophic bacteria, oligotrophic bacteria, and actinobacteria. The growth of copiotrophic bacteria was intensified in treatments with the addition of biochar and molecular sieve. The tested sorbents inhibited the proliferation of fungi whose IF_s_ values ranged from 0.205 (molecular sieve, zinc dose of 300 mg Zn^2+^ kg^−1^ DM soil) to 1.105 (halloysite, zinc dose of 900 mg Zn^2+^ kg^−1^ DM soil). Despite the above, the molecular sieve was most effective in stimulating the growth of soil-dwelling microorganisms.

**Fig. 4:**
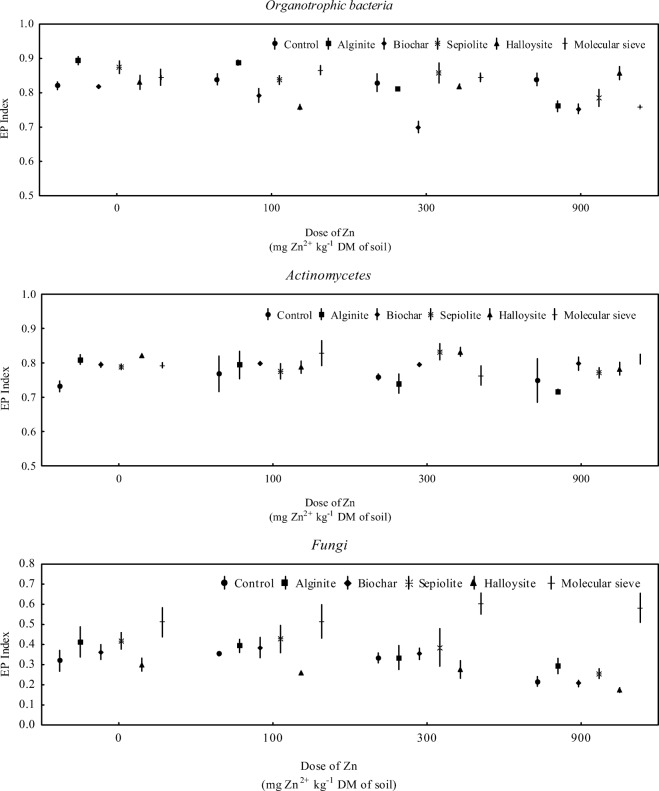
Ecophysiological diversity (EP) of microorganisms in zinc-contaminated soil. *n* = 3; points, mean value; lines, standard deviation

Soil contamination with zinc not only led to changes in microbial counts, but also affected the diversity of soil-dwelling microorganisms (Fig. 4). Fungal diversity was most compromised—the value of the EP index determined for fungi decreased from 0.319 to 0.217 under exposure to the highest zinc dose of 900 mg Zn^2+^ kg^−1^ DM soil. The influence of zinc on the EP index of organotrophic bacteria and actinobacteria was more ambiguous. In zinc-contaminated treatments, EP values were determined in the range of 0.820 to 0.839 for organotrophic bacteria, and 0.732 to 0.768 for actinobacteria. Sorbents significantly modified the EP index of soil-dwelling microorganisms. In treatments with the highest zinc dose (900 mg Zn^2+^ kg^−1^ DM soil), the application of all of the tested sorbents, excluding calcined halloysite, decreased the EP values of organotrophic bacteria. The EP index of actinobacteria exposed to the same zinc dose increased only in soil treated with the molecular sieve. The remaining sorbents did not induce significant changes in the EP index of actinobacteria after the application of a zinc dose of 900 mg Zn^2+^ kg^−1^. Unlike in organotrophic bacteria and actinobacteria, the applied sorbents significantly influenced the EP values of fungi. The molecular sieve increased the EP index of fungi relative to treatments without this sorbent. A reverse relationship was noted in treatments with the addition of halloysite. A decrease in the EP index of fungi was noted relative to treatments where sorbents were not applied. The highest EP values were observed in organotrophic bacteria, and the lowest EP values were noted in fungi.

### Biochemical Properties of Soil

The biochemical properties of soil are determined by the soil microbiome, and therefore they are reliable indicators of soil contamination, including with zinc. The activity of soil enzymes was influenced mostly by zinc dose and the applied sorbents (Table 2). Zinc dose had the greatest impact on dehydrogenase activity (50.35%), and the applied sorbent—on urease activity (79.84%). The greatest variations in catalase activity (17.18%) were observed between analytical dates.

**Table 2:** Percentage contribution of the experimental factors to the observed variation (*η*^2^) in enzyme activity in zinc-contaminated soil

Factors	Deh	Cat	Pac	Pal	Ure	Glu
Dose of Zn (a)	50.35	37.27	21.39	32.23	4.66	22.69
Sorbents (b)	25.18	7.41	32.20	52.70	79.84	32.21
Time (c)	11.08	17.18	12.47	0.18	1.11	6.87
a · b	6.45	13.98	21.46	7.68	11.47	19.67
b · c	0.86	3.91	0.52	0.75	0.45	6.39
b · c	3.89	5.98	5.04	1.05	1.43	3.53
a · b · c	2.01	12.39	6.80	5.15	0.61	8.36
Error	0.18	1.88	0.12	0.26	0.43	0.28

*Zn* zinc, *Deh* dehydrogenases, *Cat* catalase, *Pac* acid phosphatase, *Pal* alkaline phosphatase, *Ure* urease, *Glu* β-glucosidase

**Fig. 5:**
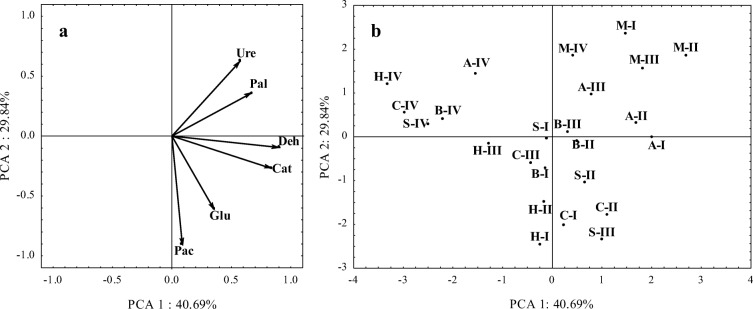
Enzyme activity in zinc-contaminated soil in principal component analysis (PCA). **a** Projection of vectors onto a plane describing enzymes activity. **b** Projection of cases onto the plane. Deh dehydrogenases, Cat catalase, Pac acid phosphatase, Pal alkaline phosphatase, Ure urease, Glu β-glucosidase, dose of zinc (mg Zn^2+^ kg^−1^ DM soil): I 0, II 100, III 300, IV 900; sorbents: C control, A alginite, B biochar, S sepiolite, H halloysite, M molecular sieve

The evaluated enzymes were divided into three groups based on their responses to the experimental factors. The first group consisted of urease and alkaline phosphatase, the second group included dehydrogenases and catalase, and the third group comprised β-glucosidase and acid phosphatase (Fig. 5a). The projection of cases revealed that the activity of dehydrogenases and catalase decreased with a rise in zinc contamination levels. Alginate and biochar stimulated the activity of oxidoreductases. The activity of alkaline phosphatase and urease was stimulated by the molecular sieve and alginate. The activity of acid phosphatase and β-glucosidase was highest in treatments without sorbents and in treatments amended with sepiolite (Fig. 5b).

**Fig. 6:**
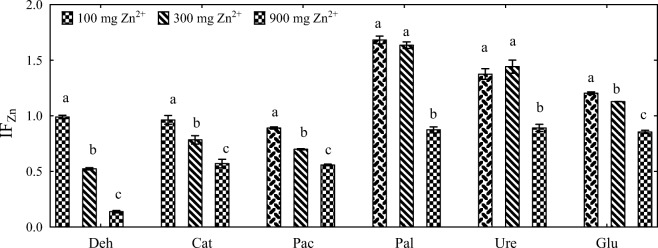
Influence of zinc (IF_Zn_) on the activity of soil enzymes in treatments without sorbents (mean values independent of time). Deh dehydrogenases, Cat catalase, Pac acid phosphatase, Pal alkaline phosphatase, Ure urease, Glu β-glucosidase. Homogeneous groups (a–c) were created separately for each enzyme (*n* = 9; *p* = 0.05)

The values of IF_Zn_ confirmed that zinc exerted varied effects on the biochemical properties of soil (Fig. 6). The activity of dehydrogenases, catalase, and acid phosphatase decreased with increasing zinc doses, particularly in treatments with the highest zinc dose (900 mg Zn^2+^ kg^−1^ DM soil). The activity of alkaline phosphatase, urease, and β-glucosidase was stimulated by zinc doses of 100 and 300 Zn^2+^ kg^−1^ DM soil. Dehydrogenases were most sensitive to high concentrations of zinc.

**Fig. 7:**
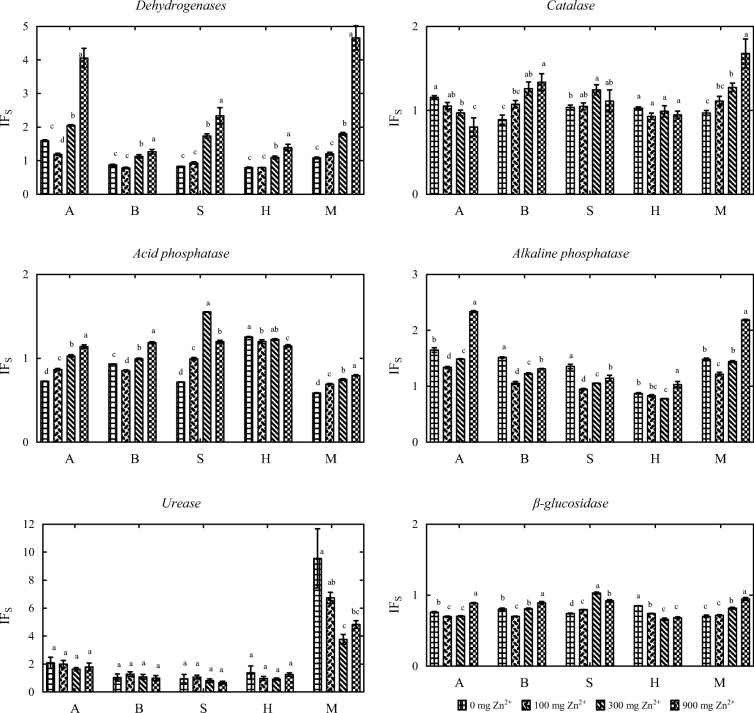
Influence of sorbents (IF_s_) on the activity of soil enzymes (mean values independent of time). A alginite, B biochar, S sepiolite, H halloysite, M molecular sieve. Dose of zinc 0, 100, 300, and 900 mg Zn^2+^ kg^−1^ DM soil. Homogeneous groups (a–d) were created separately for each enzyme and each sorbent (*n* = 12; *p* = 0.05)

In treatments not contaminated with zinc (Fig. 7), alginate and molecular sieve enhanced the activity of urease (IF_s_ of 2.025 and 9.216, respectively) and alkaline phosphatase (IF_s_ = 1.649 and 1.484, respectively). Biochar and sepiolite stimulated the activity of alkaline phosphatase (IF_s_ = 1.515 and 1.354, respectively), and halloysite increased the activity of acid phosphatase (IF_s_ = 1.254) and urease (IF_s_ = 1.308). The tested sorbents were less effective stimulants of dehydrogenases, catalase, and β-glucosidase. The activity of dehydrogenases was stimulated only by alginate (IF_s_ = 1.596). The values of IF_s_ indicate that the tested sorbents exerted varied effects on soil biochemical activity. The molecular sieve stimulated the activity of dehydrogenases, catalase, alkaline phosphatase, and urease. Alginate enhanced the activity of dehydrogenases, alkaline phosphatase, and urease. In treatments contaminated with zinc doses of 300 and 900 mg Zn^2+^ kg^−1^ DM soil, biochar and sepiolite inhibited dehydrogenase activity. Halloysite was an effective stimulant of acid phosphatase, and it also increased the activity of dehydrogenases and urease in treatments contaminated with the highest zinc dose (900 mg Zn^2+^ kg^−1^ DM soil). None of the sorbents were effective stimulants of β-glucosidase activity whose IF_s_ values ranged from 0.661 (halloysite, zinc dose of 300 mg Zn^2+^ kg^−1^ DM soil) to 1.031 (sepiolite, zinc dose of 300 mg Zn^2+^ kg^−1^ DM soil).

**Fig. 8:**
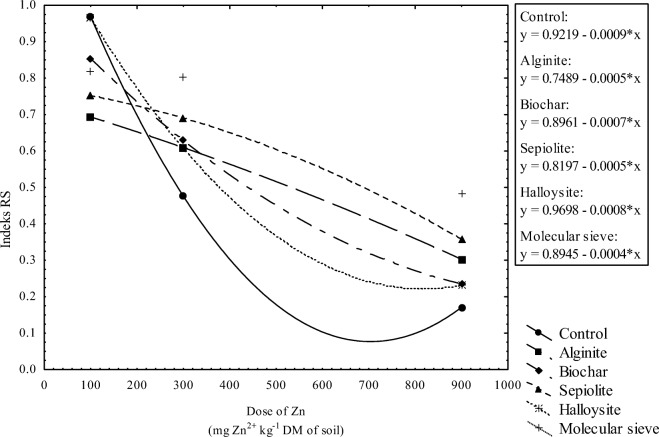
Soil resistance (RS) to zinc contamination expressed by changes in the values of the soil biochemical quality index (BA)

The values of the RS index, calculated based on the BA index, indicate that enzyme activity decreased with a rise in zinc contamination (Fig. 8). In treatments exposed to a zinc dose of 100 mg Zn^2+^ kg^−1^ DM soil, the applied sorbents decreased enzyme resistance to zinc. However, the tested remediation agents exerted protective effects in treatments contaminated with zinc doses of 300 and 900 mg Zn^2+^ kg^−1^ DM soil. The molecular sieve was most effective in minimizing the adverse effects of zinc on soil biochemical activity.

### Physicochemical and Chemical Properties of Soil

**Fig. 9:**
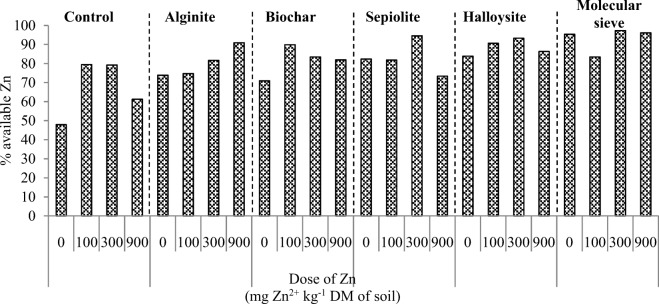
Percentage content of bioavailable zinc relative to the total zinc content of soil

The results of this study indicate that the application of zinc and sorbents induced changes in the basic physicochemical properties of soil (Table 3). In treatments contaminated with zinc, soil pH and total exchangeable bases decreased with a rise in zinc dose, which contributed to an increase in hydrolytic acidity. Soil sorption capacity and base saturation decreased in response to high zinc concentrations. In treatments exposed to the highest zinc dose (900 mg Zn^2+^ kg^−1^ DM soil), soil sorption capacity decreased by 13.5% and base saturation decreased by 15.8% relative to the control sample. The analyzed remediation agents exerted varied effects on the physicochemical properties of soil. Alginate, sepiolite, and molecular sieve significantly increased soil pH and total exchangeable bases. The remaining sorbents had a minor effect on the evaluated physicochemical parameters of soil. However, hydrolytic acidity decreased in response to all sorbents, excluding halloysite. Alginate, sepiolite, and molecular sieve induced the greatest changes. The tested sorbents increased soil sorption capacity and base saturation relative to treatments without sorbents. The applied sorbents did not decrease the concentrations of bioavailable zinc in soil (Fig. 9). In the most heavily contaminated treatments, sorbents increased the content of plant-available zinc relative to the applied zinc dose. In treatments with a zinc dose of 900 mg Zn^2+^ kg^−1^, bioavailable zinc content increased relative to control soil as follows: 1.5-fold after the application of alginite, 1.4-fold after the application of biochar and halloysite, 1.2-fold after the application of sepiolite, and 1.6-fold after the application of molecular sieve. The molecular sieve contributed to the highest increase in bioavailable zinc content relative to soil without sorbents.

**Table 3:** Physicochemical properties of soil contaminated with zinc and amended with sorbents

Dose of Zn (mg Zn^2+^ kg^−1^)	Control	Alginite	Biochar	Sepiolite	Halloysite	Molecular sieve
	pH_KCl_					
0	6.30^a^	7.13^a^	6.65^a^	7.23^a^	6.47^a^	7.42^a^
100	6.20^b^	7.10^ab^	6.63^a^	7.00^b^	6.45^ab^	7.35^b^
300	6.13^c^	7.02^b^	6.45^b^	6.90^c^	6.37^b^	7.32^b^
900	5.77^d^	6.87^c^	6.30^c^	6.60^d^	5.95^c^	7.15^c^
	Hydrolytic acidity (HAC) mM(+) kg^−1^ DM soil					
0	14.50^b^	7.50^c^	12.25^c^	9.00^c^	15.00^c^	8.50^b^
100	15.00^b^	9.00^b^	12.75^c^	10.25^bc^	16.00^bc^	9.50^ab^
300	15.50^b^	9.50^b^	14.50^b^	11.50^ab^	16.50^b^	10.00^ab^
900	19.00^a^	11.25^a^	16.25^a^	12.75^a^	19.50^a^	11.00^a^
	Total exchangeable bases (TEB) mM(+) kg^−1^ DM soil					
0	46.00^a^	125.33^a^	50.67^a^	100.67^b^	50.00^a^	122.67^a^
100	40.67^b^	123.33^a^	47.33^b^	105.33^a^	44.00^b^	118.67^b^
300	38.67^b^	120.67^a^	46.67^b^	102.67^ab^	40.00^c^	117.33^b^
900	33.33^c^	114.00^b^	44.00^c^	92.67^c^	30.67^d^	113.33^c^
	Cation exchange capacity (CEC) mM(+) kg^−1^ DM soil					
0	60.50^a^	132.83^a^	62.92^a^	109.67^b^	65.00^a^	131.17^a^
100	55.67^b^	132.33^a^	60.08^a^	115.58^a^	60.00^b^	128.17^ab^
300	54.17^bc^	130.17^ab^	61.17^a^	114.17^a^	56.50^c^	127.33^ab^
900	52.33^c^	125.25^b^	60.25^a^	105.42^b^	50.17^d^	124.33^b^
	Base saturation (BS) %					
0	76.04^a^	94.35^a^	80.53^a^	91.79^a^	76.92^a^	93.52^a^
100	73.05^ab^	93.20^ab^	78.77^a^	91.13^a^	73.34^b^	92.59^ab^
300	71.34^b^	92.70^b^	76.30^b^	89.93^b^	70.80^c^	92.15^ab^
900	63.69^c^	91.02^c^	73.03^c^	87.91^c^	61.12^d^	91.16^b^

*Zn* zinc. Homogeneous groups (a–d) were created separately for the physicochemical properties of soil and the applied sorbents (*n* = 12; *p* = 0.05)

### Maize Yield

**Fig. 10:**
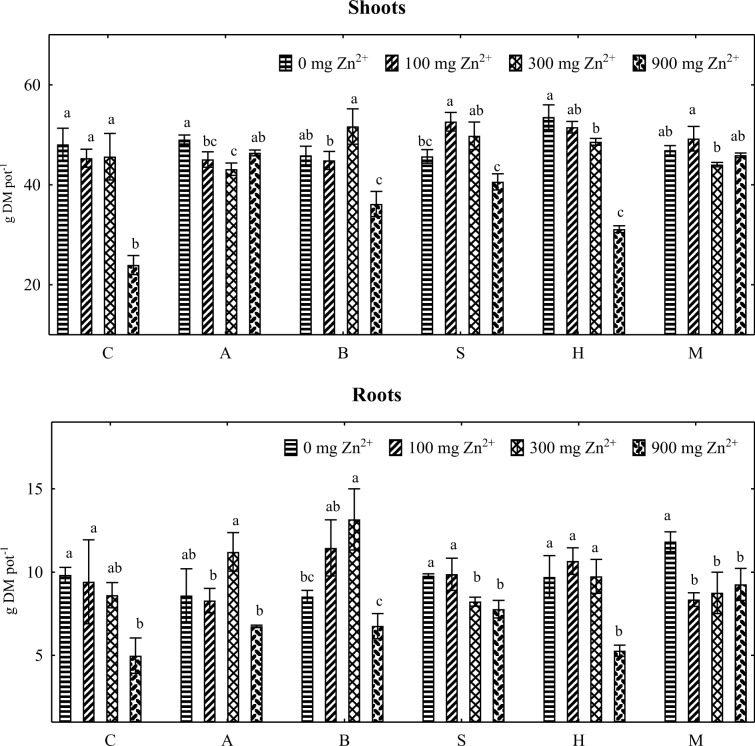
Dry matter yield of maize (g pot^−1^). C control, A alginite, B biochar, S sepiolite, H halloysite, M molecular sieve. Homogeneous groups (a–c) were created separately for each sorbent (*n* = 12; *p* = 0.05)

**Fig. 11:**
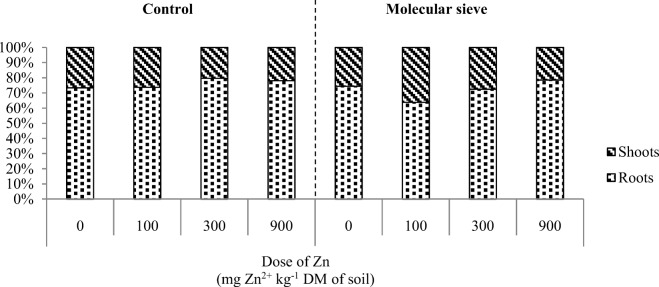
Zinc distribution in maize dry matter (%)

Zinc contamination induced significant changes in the yield of the aerial parts and roots of maize plants (Fig. 10). In treatments without sorbents, the highest zinc dose (900 mg Zn^2+^ kg^−1^ DM soil) decreased the yield of aerial plant parts by 69.04% and root yield—by 35.66%. Sorbents minimized the adverse effects of zinc on maize yield. In treatments contaminated a zinc dose of 900 mg Zn^2+^ kg^−1^ DM soil, the yield of aerial plant parts increased by 87.32% in response to alginate, 47.10% in response to biochar, 55.80% in response to sepiolite, 30.80% in response to halloysite, and 40.58% in response to the molecular sieve. The analyzed sorbents also contributed to a significant increase in root yield. In treatments with the highest zinc dose, root yield increased 2.2-fold in response to alginate, 2.8-fold in response to biochar, 2.5-fold in response to sepiolite, 1.67-fold in response to halloysite, and 34-fold in response to the molecular sieve. The molecular sieve decreased zinc uptake by the roots and increased zinc uptake by the aerial parts of maize plants (Table 4). Despite the above, the molecular sieve did not induce changes in zinc accumulation patterns in plants (Fig. 11). The zinc content of plants was similar in treatments without sorbents and in treatments containing the molecular sieve. Zinc concentrations were higher in the roots than in the aerial parts of maize plants (Fig. 10). Plants exposed to various zinc doses did not differ in their zinc accumulation patterns.

**Table 4:** Zinc content of maize plants in treatments without sorbents and in treatments where a molecular sieve was applied

	Dose of Zn (mg Zn^2+^ kg^−1^)	Control	Molecular sieve	
		mg Zn^2+^ kg^−1^		Decrease (%)
Shoots	0	23.00	21.00	8.70
	100	106.40	40.80	61.65
	300	293.00	59.20	79.80
	900	1416.00	107.20	92.43
Roots	0	63.60	61.00	4.09
	100	299.00	72.00	75.92
	300	1148.00	156.00	86.41
	900	5030.00	392.00	92.21

## Discussion

The soil microbiome can be modified under exposure to external factors, including substances with toxic effects. In the present study, zinc intensified the proliferation of soil-dwelling microorganisms. Microbial growth was determined by both the zinc dose and the date of analysis. The stimulatory effects of zinc on the abundance of soil microorganism have also been observed by other authors Wyszkowska et al. (2013). However, excessive zinc concentrations (1200 mg Zn^2+^ kg^−1^ DM soil) can disrupt the soil microbiome and reduce microbial abundance (Wyszkowska et al. 2016). According to Aiju et al. (2013), the zinc content of soil of 74.33 to 127.20 mg kg^−1^ may lead to adverse changes in the microbiological parameters of soil ecosystems. Kouchou et al. (2017) noted a decrease in the counts of fungi and actinobacteria and an increase in bacterial counts in soil containing 291.20 mg Zn^2+^ kg^−1^, compared with control soil. In the present study, the highest zinc dose (900 mg Zn^2+^ kg^−1^ DM soil) exerted a toxic effect on organotrophic bacteria and decreased the counts of the remaining microbial groups in treatments contaminated with zinc doses of 100 and 300 mg Zn^2+^ kg^−1^ DM soil. The above zinc doses also induced changes in the EP index of soil-dwelling microorganisms. Zinc applied at the highest dose (900 mg Zn^2+^ kg^−1^) could exert a toxic effect on soil-dwelling microorganisms, which was reflected in changes in microbial diversity. Excessive zinc concentrations in soil made it impossible for the microorganisms to adapt to the existing conditions. When present in high concentrations, the heavy metal could penetrate the cell membranes of microorganisms, leading to changes in the structure and function of cell organelles, followed by cell death. The observed changes in biodiversity could also result from microbial succession. The soil environment was dominated by microbial species resistant to high zinc concentrations, which eliminated the remaining microorganisms. The negative effect of zinc on soil biological activity was also observed by Epelde et al. (2010) who found that a zinc dose of 1000 mg kg^−1^ inhibited the proliferation of total bacteria and nitrifying bacteria. Other authors also reported a decrease in the counts of organotrophic bacteria, bacteria of the genus *Azotobacter*, actinobacteria, and fungi in soil contaminated with zinc doses of 150 to 1200 mg Zn^2+^ kg^−1^ DM soil (Wyszkowska et al. 2015). According to Macdonald et al. (2008), long-term application of heavy metals, including zinc, may lead to changes in the proliferation and structure of microorganisms. Zinc doses of 150–450 mg Zn^2+^ kg^−1^ significantly affected the structure of microbial communities, in particular fungi.

Changes in the microbiological properties of soil also disrupt soil biochemistry and decrease enzyme activity (Borowik et al. 2014; Wieczorek et al. 2014; Wyszkowska et al. 2016; Yang et al. 2006). Soil enzymes rapidly respond to contamination, and they are good indicators of the changes induced by xenobiotics. In the current study, zinc inhibited the activity of all analyzed soil enzymes (dehydrogenases, catalase, urease, acid phosphatase, alkaline phosphatase, β-glucosidase). The highest zinc dose (900 mg Zn^2+^ kg^−1^ DM soil) had a particularly inhibitory influence on enzyme activity. Similar results were reported in a study evaluating the influence of zinc doses of 150, 300, 600, and 1200 mg Zn^2+^ kg^−1^ DM soil on the activity of dehydrogenases, urease, acid, and alkaline phosphatase (Boros et al. 2011). The cited authors observed that enzyme activity decreased with increasing zinc levels. According to Borowik et al. (2014), zinc doses of 300 to 2400 mg Zn^2+^ kg^−1^ DM soil can suppress enzyme activity in soil. In the work of Kucharski et al. (2011), soil enzymes were arranged in the following order based on their sensitivity to zinc contamination: arylsulfatase > dehydrogenases > acid phosphatase > urease > β-glucosidase.

Excess zinc can also compromise plant growth and development. In our study, zinc doses of 100 to 900 mg Zn^2+^ kg^−1^ DM soil decreased the yield of maize roots and aerial parts.

Substances with soil remediation potential are increasingly used to limit the negative consequences of soil contamination with trace elements. These substances decrease the mobility of heavy elements by modifying soil properties such as pH, content of clay minerals and organic matter, cation-exchange capacity, and soil type (Bradl 2004). Other remediation substances promote microbial growth, intensify the biological activity of soil and, consequently, improve soil fertility. In our study, the tested sorbents, in particular alginate and biochar, contributed to the proliferation of organotrophic bacteria, oligotrophic bacteria, copiotrophic bacteria, and actinobacteria. Fungal counts decreased in response to the applied soil remediation agents. The application of sorbents could lead to the immobilization of soil microorganisms, thus inhibiting their proliferation and soil enzymatic activity. The presence of mutual interactions between soil-dwelling microorganisms and biochar was reported by Lehmann and Rondon (2006). Biochar can modify habitat conditions, whereas microorganisms can alter the quantity and properties of biochar. Biochar is characterized by high adsorption capacity (Liang et al. 2006) and a large specific surface area which creates supportive conditions for the immobilization of soil microorganisms (Lehmann et al. 2011). In treatments not contaminated with zinc, the application of biochar increased the EP values of microorganisms. In a study by Steiner et al. (2008), biochar also stimulated the soil microbiome by increasing microbial biomass and microbial activity assessed based on microbial respiration. Biochar increases the bioavailability of soil nutrients and soil pH, which not only contributes to crop yields, but also enables crop production in previously degraded areas (Lehmann and Rondon 2006). In a study evaluating the influence of sepiolite on the soil environment, the analyzed mineral stimulated the proliferation of bacteria and actinobacteria (Sun et al. 2013). In our study, sepiolite did not induce significant changes in the counts of the analyzed microbial groups. Calcined halloysite was not highly effective in improving the microbiological properties of soil. In the work of Wyszkowska et al. (2015), cellulose and fermented bark alleviated the negative effects of zinc on the abundance of *Azotobacter* bacteria, organotrophic bacteria, and actinobacteria, whereas keratin stimulated fungal growth.

The analysis of enzyme activity in soil also revealed that the applied sorbents minimized the toxic effects of zinc. The evaluated sorbents stimulated the activity of dehydrogenases and catalase which are good indicators of soil microbial activity. In treatments remedied with alginate, the above effects can be attributed to increased organic matter content (Sheng et al. 2004). According to Moreno et al. (2009), enzyme activity is less compromised by zinc in soils with higher organic matter content. Extracellular enzymes are immobilized or form complexes with organic matter and exert protective effects (Kızılkaya et al. 2004). The addition of organic matter to soil stimulates microbial proliferation and increases the biodiversity and activity of enzymes, thus minimizing the adverse effects of zinc (Strachel et al. 2017). Organic matter contains humic substances which play an important role in the environment due to their affinity for soil contaminants (Szabó 2004). Sheng et al. (2004) demonstrated that algae and algal derivatives were effective in decreasing the bioavailability of heavy metals. These effects can be attributed to the functional groups of cell wall polysaccharides. In the work of Tica et al. (2011), an alginate-based substance with high adsorption capacity increased soil pH. Humic substances also increase heavy metal uptake by plants, which is an important consideration in the process of soil phytoremediation. Humic substances and heavy metals form stable complexes, which decreases the mobility of toxic compounds in the soil solution (Halim et al. 2003). In a study by Boros et al. (2011), cellulose and fermented bark stimulated enzyme activity in soil, whereas keratin inhibited the activity of all evaluated enzymes (dehydrogenase, urease, acid, and alkaline phosphatase) and did not alleviate the toxic effects of zinc. In the current study, calcined halloysite stimulated catalase activity. According to Zhai et al. (2010), halloysite contributed to enzyme stability under adverse environmental conditions. Similarly to sepiolite, halloysite is a clay mineral with a large specific surface area which effective absorbs pollutants, including heavy metals (Wu et al. 2016). Halloysite binds trace elements through cation exchange or the formation of surface complexes (Yuan et al. 2015). In addition, materials with high adsorption capacity regulate the microbiological and biochemical properties of soil. According to Lehmann et al. (2011), these substances contribute to the retention of water and nutrients in soil and immobilize microorganisms on their surface. In the work of Sun et al. (2016), bentonite, which is also a clay mineral, exerted protective effects on the soil microbiome and soil enzymes. Bentonite increased the counts of actinobacteria and stimulated catalase activity. In our study, sepiolite induced changes in the activity of alkaline phosphatase and β-glucosidase. The beneficial effects of sepiolite were also reported in a study by Sun et al. (2013) where this mineral enhanced the activity of urease and invertase. Sepiolite can also contribute to increasing soil respiration and the activity of dehydrogenase and alkaline phosphatase (Abad-Valle et al. 2016). Sepiolite immobilizes heavy metals, including zinc, by replacing magnesium ions on the boundaries of octahedral magnesium layers in the substrate (De Lima et al. 2017). In our study, the molecular sieve induced the greatest increase in soil resistance (RS) to zinc, determined based on the BA index. These changes were most pronounced in treatments contaminated with the highest zinc dose (900 Zn^2+^ kg^−1^ soil DM). According to Brach et al. (2017), the presence of nanopores in the molecular sieve supports interactions with the ions and molecules of chemical compounds. The above mechanism could be responsible for the observed increase in the counts of bacteria and actinobacteria and enzyme activity. These observations also suggest that substances with a large specific surface area exert protective effects by immobilizing microorganisms and enzymes on their surface.

Changes in the microbiological properties of soil and enzyme activity influence the rate of metabolic processes and, consequently, the physicochemical properties of soil. In our study, the applied sorbents did not decrease the content of bioavailable zinc relative to total zinc concentration in soil. It was found that the molecular sieve decreased zinc uptake by the roots and increased zinc uptake by the aerial parts of maize plants. The tested sorbents increased maize yield, in particular in the most highly contaminated treatments. Our results suggest that the content of bioavailable zinc in soil was significantly modified by nutrient cycling and root secretions. The analyzed soil remediation substances minimized the toxic effects of zinc mainly by promoting the growth of soil-dwelling microorganisms, including organotrophic bacteria, oligotrophic bacteria, and actinobacteria, and by stimulating most soil enzymes, which contributed to an increase in maize yield.

## Conclusions

Increasing levels of environmental pollution prompt the search for new solutions to guarantee global food security. In the described experiment, high zinc doses deteriorated the soil environment by decreasing the counts and biodiversity of microorganisms and disrupting biochemical processes in soil. Zinc had an adverse effect on the growth and development of maize, and it decreased the yield of aerial plant parts. Remediation substances capable of absorbing pollutants were applied to limit the adverse influence of zinc on the soil environment. The tested sorbents generally minimized the toxic effects of zinc on soil microorganisms and enzymes. The analyzed substances also induced changes in the microbiological diversity of soil. The evaluated sorbents did not limit the bioavailability of zinc in soil, but decreased the accumulation of zinc in maize plants. Our findings indicate that not all of the tested sorbents can be effectively used to remediate zinc-contaminated soil. The greatest increase in soil fertility was observed under the influence of alginate, biochar, and molecular sieve. The molecular sieve emerged as the most effective substance for the remediation of soil contaminated with zinc.
